# Global Analysis of the Genetic Variations in miRNA-Targeted Sites and Their Correlations With Agronomic Traits in Rapeseed

**DOI:** 10.3389/fgene.2021.741858

**Published:** 2021-09-14

**Authors:** Pengfei Xu, Yantao Zhu, Yanfeng Zhang, Jianxia Jiang, Liyong Yang, Jianxin Mu, Xiang Yu, Yuke He

**Affiliations:** ^1^National Key Laboratory of Plant Molecular Genetics, CAS Center for Excellence in Molecular Plant Sciences, Shanghai Institute of Plant Physiology and Ecology, Chinese Academy of Sciences (CAS), Shanghai, China; ^2^University of the Chinese Academy of Sciences, Beijing, China; ^3^Hybrid Rape Research Center of Shaanxi Province, Yangling, China; ^4^Crop Breeding and Cultivation Research Institute, Shanghai Academy of Agricultural Sciences, Shanghai, China; ^5^School of Life Sciences and Biotechnology, Shanghai Jiao Tong University, Shanghai, China

**Keywords:** rapeseed, miRNA target site, genetic variant, agronomic trait, correlation analysis

## Abstract

MicroRNAs (miRNAs) and their target genes play vital roles in crops. However, the genetic variations in miRNA-targeted sites that affect miRNA cleavage efficiency and their correlations with agronomic traits in crops remain unexplored. On the basis of a genome-wide DNA re-sequencing of 210 elite rapeseed (*Brassica napus*) accessions, we identified the single nucleotide polymorphisms (SNPs) and insertions/deletions (INDELs) in miRNA-targeted sites complementary to miRNAs. Variant calling revealed 7.14 million SNPs and 2.89 million INDELs throughout the genomes of 210 rapeseed accessions. Furthermore, we detected 330 SNPs and 79 INDELs in 357 miRNA target sites, of which 33.50% were rare variants. We also analyzed the correlation between the genetic variations in miRNA target sites and 12 rapeseed agronomic traits. Eleven SNPs in miRNA target sites were significantly correlated with phenotypes in three consecutive years. More specifically, three correlated SNPs within the miRNA-binding regions of *BnSPL9-3*, *BnSPL13-2*, and *BnCUC1-2* were in the loci associated with the branch angle, seed weight, and silique number, respectively; expression profiling suggested that the variation at these 3 miRNA target sites significantly affected the expression level of the corresponding target genes. Taken together, the results of this study provide researchers and breeders with a global view of the genetic variations in miRNA-targeted sites in rapeseed and reveal the potential effects of these genetic variations on elite agronomic traits.

## Introduction

Traditional crop breeding requires phenotypic evaluations and its success is mainly dependent on the experience of the breeder. The recent development of molecular breeding by design has led to increased breeding efficiency (Zeng et al., 2017). On the basis of advances in functional genomic research and genetic studies on families or natural populations, a large number of single nucleotide polymorphisms (SNPs) contributing to agriculturally important traits have been identified. Re-sequencing followed by SNP genotyping is one of the key strategies used for developing molecular markers for specific agronomic traits (Morrell et al., 2012). Including SNPs that change protein sequences as well as those that affect transcriptional and post-transcriptional regulation involving *cis*-elements is also important. For example, SNPs in gene promoters targeted by transcription factors regulate transcription, whereas functional SNPs in exon–intron junctions, protein–RNA binding sites, and microRNA (miRNA)-targeted sites contribute to post-transcriptional regulation (Zhang W. et al., 2018). Therefore, these functional SNPs are key genetic resources for crop breeding.

MicroRNAs, which are small RNAs comprising 20–24 nucleotides, function as important post-transcriptional regulators of gene expression in plants and animals (Bartel, 2004; Dugas and Bartel, 2004; Kidner and Martienssen, 2005; Chuck et al., 2009). Plant miRNAs are generated from stem-loop precursors by the DCL1 complex. Mature miRNAs are loaded into the RNA-induced silencing complex to enable AGO1 to cleave mRNAs complementary to specific miRNAs, and their miRNA^∗^ strand is subsequently degraded (Shukla et al., 2008; Chen et al., 2016). Inhibited gene expression via the translational arrest mediated by the AGO1 complex is also a common phenomenon among plants (Brodersen et al., 2008). Additionally, miRNAs negatively regulate the expression of specific genes by targeting the untranslated region (UTR) or the coding region of mRNAs. Several studies revealed that miRNAs and their target genes play important roles in the vegetative phase and the transition to reproductive development (Wang et al., 2009, 2014; Tripathi et al., 2018), signal transduction pathways (Navarro et al., 2006; Liu et al., 2009; Inui et al., 2010), plant morphogenesis (Liu et al., 2010, 2019; Mao et al., 2014; Ren et al., 2018), and biotic and abiotic stress responses (Lu et al., 2013; Li et al., 2019). For example, miR156 is conserved among species and affects shoot branching and plant architecture by regulating the expression of *SPL* genes (Wang et al., 2009; Ferreira e Silva et al., 2014; Liu et al., 2017). The overexpression of miR156 affects several traits, including plant height, flowering time, leaf number, root length, grain yield, and branch number (Wang et al., 2009; Wang and Zhang, 2017). Thus, miR156–*SPL* modules coordinately regulate diverse traits in crops.

MicroRNAs control many biological processes by silencing target genes. Moreover, they have been broadly applied for manipulating eukaryotic gene expression. The variability of the DNA sequences at miRNA target sites alters the complementation between miRNAs and their target genes, which influences the target gene expression levels and the traits relevant to these genes (Mallory et al., 2004). However, the extent of the genetic variations at miRNA target sites and the agronomic traits that are altered by the variations remains unclear.

Rapeseed (*Brassica napus*) is one of the most important oilseed crops in the genus Brassica. It is mainly grown in Europe, Asia, North America, and Oceania, where it is an important oilseed crop. Through natural and artificial selection, rapeseed has become a multipurpose crop (i.e., vegetable crop, forage crop, and oilseed crop) (Allender and King, 2010). It contains two divergent sub-genomes, A and C, which are shaped by a whole-genome triplication followed by extensive diploidization (Parkin et al., 2005; Mandáková and Lysak, 2008). Thus, its genome structure is complex, which has hindered genomic research and the high-throughput identification of high-quality molecular markers. Researchers and breeders have bred many elite rapeseed germplasms through the laborious selection of target traits. These germplasms have been exploited to breed new cultivars and have shown great potential for increasing crop yield and quality. However, the molecular mechanism underlying trait selection remains unclear. Studying the genetic variants generated during the formation of elite rapeseed germplasms may produce valuable information regarding the genetic changes, thereby facilitating the efficient selection of elite germplasms for breeding.

Current research on important agronomic traits includes the detection of global genetic variations and genome-wide association studies following by the fine-mapping of the causal genetic variants affecting a particular trait. However, in this study, we focused on elucidating the genetic variations in the miRNA-complementary region of target genes in 210 elite rapeseed accessions. We detected three SNPs in miRNA target sites that are, respectively, correlated with the branch angle, silique number, and seed weight. The results of this study provide an overview of the genetic variations in miRNA target sites as well as additional functional SNPs relevant for rapeseed molecular breeding.

## Materials and Methods

### Plant Materials

For this study, 650 rapeseed accessions were obtained from the Center for Shaanxi Hybrid Rapeseed and Shanghai Academy of Agricultural Sciences, China. On the basis of the information provided by the rapeseed accession donor and our own observations, 210 elite accessions were selected from the 650 accessions as the core collection of genetically diverse rapeseed accessions with varying phenotypic traits for the analysis of genomic variations (Supplementary Table 1). To obtain homozygous genotypes, each accession was self-fertilized for more than five generations and one representative plant of each accession was selected for DNA re-sequencing. These accessions were assigned to different germplasm categories according to their growing seasons. More specifically, the accessions were designated as winter (7), semi-winter (198), and spring (5) oilseed rape (Supplementary Table 1). The accessions originated from Asia, Europe, and North America (Supplementary Table 1). Their seeds were sown in experimental field plots at the Farm Station in Yangling, Shaanxi province, China in 2016, 2017, and 2018. The accessions were grown using a randomized complete block design with two replicates. Each accession was grown in plots with four rows, which were separated by 25 cm. The 22–25 plants in each row were separated by 8–12 cm. Between 5 and 10 representative plants in the middle of each plot were selected at harvest to analyze the plant height, silique number, seed number, seed weight, oil content, branch number, and branch angle. The flowering time, ripening time, cold resistance, *Sclerotinia sclerotiorum* resistance, and stem lodging resistance were analyzed in the field at a representative stage. The average trait values of the inbred line replicates in each year were used for phenotypic and correlation analyses in a single environment. The correlations among traits were evaluated using the *cor.test* function in R (version 3.6.1).

### DNA Re-sequencing and Alignment With the Reference Genome

Developing leaves were collected from individual plants for a genomic DNA extraction using the Plant Genomic DNA Kit (Tiangen, Beijing, China). The concentration and quality of the extracted genomic DNA were determined using the NanoDrop 2000 spectrophotometer (Thermo Fisher Scientific). The DNA samples with an OD_260_/OD_280_ ratio of 1.8–2.0 and a total content of more than 1.5 μg were suitable for constructing sequencing libraries. An Illumina sequencing library was constructed for each accession using the IlluminaTruSeq Library Construction Kit. Paired-end sequencing was performed using the Illumina HiSeq platform, with a read length of 150 bp at each end (Novogene, Tianjin, China). Raw reads were filtered according to the following criteria: paired-end reads with >10% “N” bases and Q ≤ 10. The clean reads for each accession were mapped to the *B. napus* “Darmor” reference genome (version 4.1), which was downloaded from BRAD^1^ (Chalhoub et al., 2014). The PCR-duplicated reads were removed by Picard MarkDuplicates. The SNPs and INDELs were called for all 210 *B. napus* accessions using GATK.^2^ The identified variants were annotated using ANNOVAR.^3^ Additionally, AGAT-master was used to convert the gff3 file to a gtf file^4^.

### Predication of miRNA-Complementary Target Regions

The miRNA target sites were predicted using psRNATarget,^5^ with default parameters and a maximum expectation value of 3.5. The targets were all protein-coding transcripts in the *B. napus* transcript library in BRAD (see text footnote 1). To ensure target sites were accurately identified, the putative target sites were further evaluated using the available data generated by degradome sequencing or the 5′ RNA ligase-mediated rapid amplification of cDNA ends. Incorrectly predicted target sites were removed.

### Extraction of SNPs and INDELs From miRNA Target Sites and Correlation Analyses

The physical positions of target genes were obtained from the gff3 file (see text footnote 1). The BLASTN (blastn-short) algorithm was used to determine the locations of miRNA target sites within the reference genome. Variants were extracted from the genotype data using perl packages. Genotypes were filtered using PLINK2. Of the target site regions, 330 raw SNPs were detected and then a pseudo-VCF was created. The TASSEL 5 program^6^ was used to convert the VCF format to the Hapmap format. Finally, 199 high-quality SNPs (MAF > 0.05, integrity ≥ 0.7) were obtained for the single-SNP correlation analysis, which was conducted using the general linear model in the TASSEL 5 program.

### Quantitative Real-Time PCR

Total RNA was extracted from young leaves using the TRIzol reagent, after which DNase I (Takara) was used to remove DNA contaminants. The total RNA (2 μg) was reverse transcribed into cDNA using PrimeScript Reverse Transcriptase (TaKaRa) and an oligo (dT) primer. The qRT-PCR was performed using the SYBR Green Real-time PCR Master Mix (TOYOBO, Japan) and specific primer pairs (Supplementary Table 2) to quantify relative transcript levels. The samples analyzed by qRT-PCR are listed in Supplementary Table 3. The *ACTIN7* gene was used as an internal control for analyzing gene expression. The qRT-PCR was completed with three biological replicates and three technical replicates.

### Genome-Wide Association Study

GWAS was performed for each trait using a mixed linear model (MLM) in TASSEL 5 program. The SNPs detected in more than 80% of accessions and MAF > 0.05 were used. The Linkage Disequilibrium (LD) between each pair of SNPs and the LD decay values were calculated using PopLDdecay.^7^ More details about GWAS were described in previous reports (Sun et al., 2016).

### Bioinformatics Tools

The information of main Bioinformatics tools used in this study is listed in Supplementary Table 4.

## Results

### Overview of the Genetic Variations in 210 Rapeseed Accessions

**FIGURE 1:**
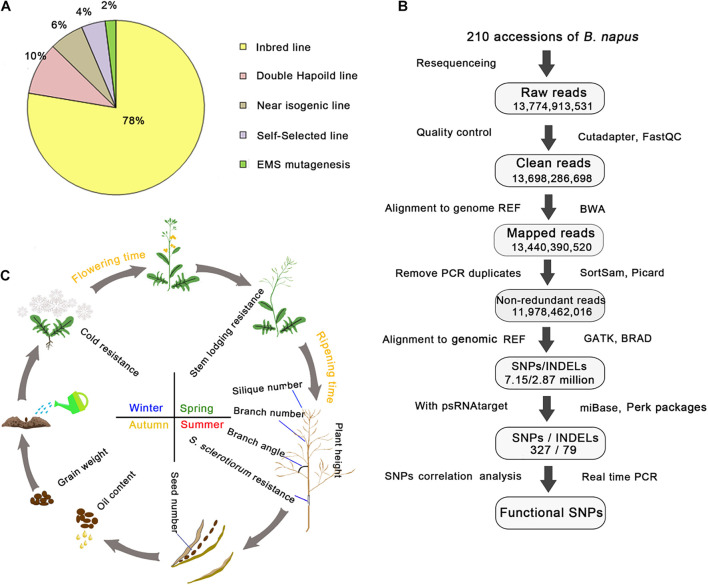
Summary of the 210 rapeseed accessions exhibiting 12 important agronomic traits and the pipeline for analyzing genetic variations. **(A)** Percentages of different rapeseed categories. **(B)** Pipeline for the DNA re-sequencing and correlation analysis. Genome REF refers to the *B. napus* “Darmor” reference genome, whereas transcript REF refers to the *B. napus* transcript reference sequences; both were downloaded from the BRAD website. The number in each frame represents the sum of the values. Cutadapter, FastQC, BWA, SortSam, Picard, GATK, and psRNATarget were the main bioinformatic tools used in the pipeline, whereas TAIR, BRAD, and miRBase were the key databases used in this study. **(C)** Schematic representation of the 12 important agronomic traits. The arrows indicate a life cycle.

To investigate the genetic variations in rapeseed, we collected 210 elite rapeseed accessions exhibiting 12 elite agronomic traits from worldwide collections, including 164 inbred lines, 20 doubled haploid lines, 14 near isogenic lines, 4 ethyl methanesulfonate-mutated lines, and 8 self-selected lines (Supplementary Table 1 and Figure 1A). All elite accessions had one or more desirable agronomic trait and were derived from interspecific crosses, mutagenesis, and natural variations (Supplementary Table 1). The analysis of re-sequencing data confirmed that almost all accessions were homozygous and suitable for further study.

The DNA re-sequencing analysis of 210 rapeseed accessions generated 2.94 Tb of raw data. After a quality control step, 14 billion high-quality clean reads were obtained. The number of clean reads per accession ranged from 56 to 86 million, with a mean of 65 million clean reads. All clean reads from each accession were aligned to the *B. napus* “Darmor” variety reference genome (version 4.1). The mapping rate ranged from 83 to 99%, with a mean of 98%, and the effective coverage depth for each line ranged from 9- to 25-fold, with a mean of 12-fold (Supplementary Table 1). Duplicated reads caused by the PCR amplification were filtered and the remaining non-redundant reads were used for genotyping. The number of non-redundant reads for each accession ranged from 40 to 74 million, with a mean of 57 million reads (Supplementary Table 5 and Figure 1B). On the basis of the alignment with the reference genome, 7.15 million SNPs and 2.89 million insertions/deletions (INDELs) were identified in the 210 rapeseed accessions. These variants were distributed on 19 chromosomes and scaffolds. Additionally, 2.01 million SNPs and 1.63 million INDELs were rare variants in these rapeseed accessions [minor allele frequency (MAF) ≤ 0.05, integrity ≥ 0.7]. Approximately 40.22% of the variants were rare events.

We subsequently annotated the genomic variants in rapeseed using ANNOVAR (Supplementary Table 6). Most of the variants were in intergenic regions (52.94% of the SNPs and 43.98% of the INDELs). Regarding the genic regions, 10.83% of the SNPs and 20.37% of the INDELs were in introns, 15.86% of the SNPs and 3.84% of the INDELs were in exons, 1.36% of the SNPs and 2.60% of the INDELs were in 3′-UTRs, and 0.85% of the SNPs and 1.94% of the INDELs were in 5′-UTRs. Moreover, 8.87% of the SNPs and 13.57% of the INDELs were in the 1 kb region upstream of the transcription start site, whereas 7.51% of the SNPs and 10.20% of the INDELs were in the 1 kb region downstream of the transcription termination site. Accordingly, these variants may affect transcription.

### Prediction of miRNA Target Sites in Rapeseed Transcripts

**FIGURE 2:**
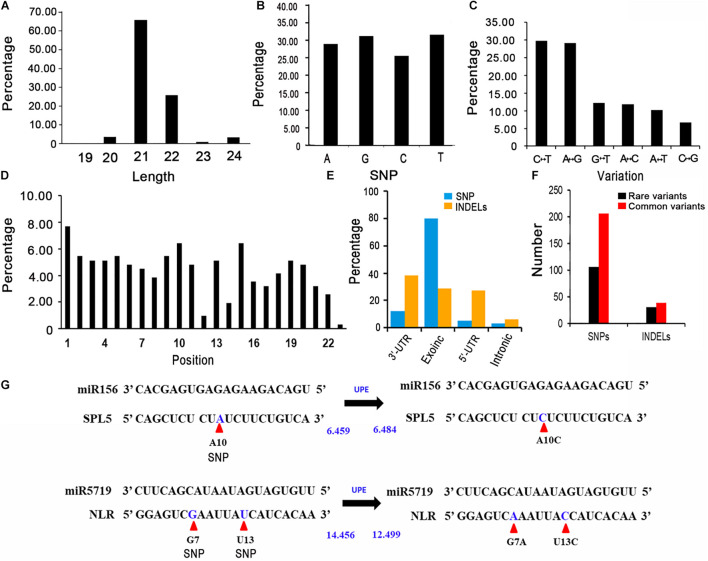
Features of the variants in miRNA target sites. **(A)** Length distribution of putative miRNA target sites. **(B)** Percentages of the A, G, C, and T variations. **(C)** Percentages of the different types of nucleotide substitutions among the SNPs. C↔T: C to T or T to A substitution; G↔A: G to A or A to G substitution; C↔T: G to T or T to G substitution; A↔C: A to C or C to A substitution; A↔T: A to T or T to A substitution; C↔G: C to G or G to A substitution. **(D)** Percentage of SNPs in each miRNA target site. **(E)** Annotation of the variants identified in miRNA target sites. **(F)** Number of rare and common variants in miRNA target sites. **(G)** Altered target binding caused by genetic variants within miRNA target sites. UPE: target accessibility energy to unpair the target site (kcal/mol); decreases in energy increase the possibility that small RNAs will be able to interact with (and cleave) target mRNAs. Allelic SNPs are presented in blue and are marked by red arrows. UPEs are calculated using psRNAtarget.

In general, miRNAs negatively regulate gene expression by targeting the 3′-UTR or the coding region of mRNAs. Therefore, variants in exons and UTRs might significantly affect gene expression via miRNA binding. To reveal the genetic variations in miRNA target sites in rapeseed, we first identified the gene regions bound by miRNAs using psRNATarget with cruciferous miRNAs, with a maximum expectation value of 3.5 (Jones-Rhoades and Bartel, 2004; Alves et al., 2009; Yu et al., 2011; Dai et al., 2018; He J. et al., 2018; Zhang X. D. et al., 2018). A total of 163 crucifer miRNAs from four species (*B. napus*, *Brassica rapa*, *Brassica oleracea*, and *Arabidopsis thaliana*) were retrieved from the miRbase database (version 22)^8^ (Supplementary Table 7). The protein-coding transcripts were downloaded from BRAD (see text footnote 1). Together with previously reported degradome data and the results of the 5′ RNA ligase-mediated rapid amplification of cDNA ends (Lu et al., 2008; Vaucheret, 2009; Chen et al., 2011; Ng et al., 2011; Kinoshita et al., 2012; Bari et al., 2013; Song et al., 2013; Zhang et al., 2013, 2015; Liu H. et al., 2014; Park et al., 2014; Windels et al., 2014; Xian et al., 2014; Ma et al., 2015, 2017; Ai et al., 2016; Ouyang et al., 2016; Leng et al., 2017; He F. et al., 2018; Kuo et al., 2018; Tang et al., 2018; Zheng et al., 2019), we identified 1,644 high-confidence target site sequences (Supplementary Table 8). After being generated from stem-loop precursors by the DCL1 complex, the mature miRNAs are loaded into an AGO1-dominant RNA-induced silencing complex (RISC) and repress the target transcripts mainly in two ways: cleavage and translational repression (Brodersen et al., 2008). For the miRNA target sites, 1284 were predicted to be cleaved and 360 could repress translation by the AGO1. In addition, 97% of the target site sequences were 20–22 nucleotides long, with the most (77%) was 21 nucleotides long (Figure 2A).

To functionally characterize the miRNA target genes in rapeseed, we annotated the protein-coding genes in *B. napus* via a BLASTN search of the Araport11 genome release in The Arabidopsis Information Resource (TAIR) database.^9^ The annotation of rapeseed genes is relatively limited, but the functions of these genes may be determined by analyzing the similarity between the mRNA sequences of rapeseed and *A. thaliana* (85% homology) (Cavell et al., 1998). In total, 101,040 protein-coding genes have been reported in rapeseed (Chalhoub et al., 2014). Here, we found 67,288 genes were homologous to *A. thaliana* genes and 33,752 genes were specific to rapeseed (Supplementary Figure 1A and Supplementary Table 9). Among the 1,644 identified miRNA target genes, 1,394 were homologous to *A. thaliana* genes, whereas 250 were specific to rapeseed (Supplementary Figure 1B).

**FIGURE 3:**
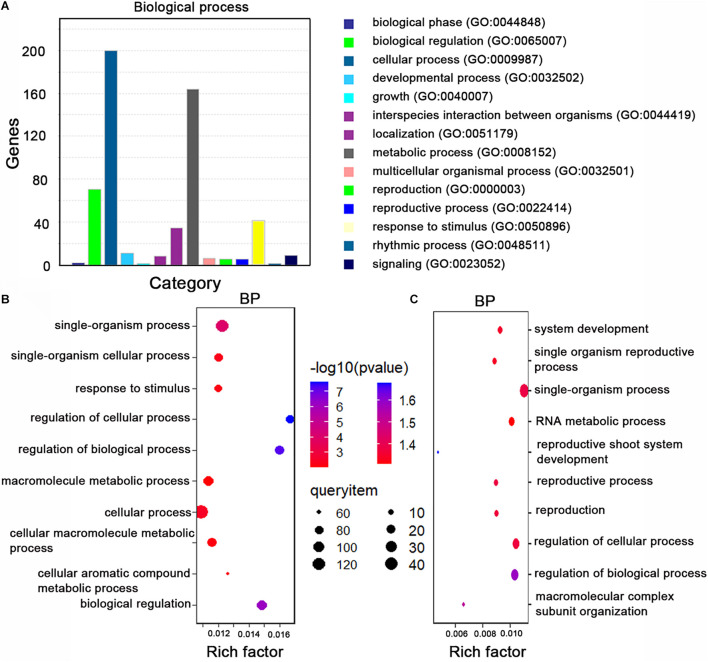
Gene ontology (GO) analyze the miRNA target genes. **(A)** The top 14 GO terms (biological process category) among the miRNA target genes. The enriched processes are listed on the right. **(B)** The top 10 GO terms (biological process category) among the 328 variant miRNA target genes in *B. napus*. The enriched processes are listed on the left. **(C)** The top 10 GO terms (biological process category) among the 124 rare variant target genes in rapeseed. The enriched processes are listed on the right. Rich factor: number of candidate genes/total number of genes. BP: biological process.

Next, we performed a gene ontology (GO) enrichment analysis of the miRNA target genes homologous to *A. thaliana* genes. The overrepresented GO terms in the biological process category were response to stimulus, metabolic process, and biological regulation (Figure 3A). The main GO terms in the molecular function category were adenyl nucleotide and ribonucleotide binding and ATP binding.

### Genetic Variations in the miRNA Target Sites in Rapeseed Accessions

To identify the genetic variations in the miRNA target sites in the examined rapeseed accessions, we extracted the variants from the genotype data. We detected 310 raw SNPs and 79 INDELs in the miRNA target sites (Supplementary Table 10). A global survey of SNPs in the miRNA target sites (integrity ≥ 0.7) revealed that 24.68, 26.60, 21.79, and 26.92% of the single-base variations resulted in an A, G, C, and T, respectively (Figure 2B). The C↔T (U) and A↔G substitutions were the most common variations, which is consistent with the fact that U/G at an mRNA site can still partially pair with G/U of a mature miRNA (Figure 2C). The SNPs were more frequent at the first, tenth, and fifteenth nucleotides of the target site than at the other nucleotides. The SNPs were the least frequently detected at the twelfth and twenty-third nucleotides (Figure 2D). To clarify the potential effects of these variants, the detected SNPs and INDELs (integrity ≥ 0.7) were functionally annotated by ANNOVAR according to the “*Darmor*” gene model. Most of the SNPs (80.12%) were in exons and 11.93% were in 3′-UTRs (Supplementary Table 6 and Figure 2E). This result was consistent with miRNAs targeting the 3′-UTR or the coding region of mRNAs. These SNPs were linked to 257 miRNA target genes, of which 214 were homologs of *A. thaliana* genes and 43 were specific to rapeseed (Supplementary Figure 1C). Most of the INDELs (94.05%) were in exons and UTRs. More specifically, 38.10, 28.57, and 27.38% of the INDELs were in the 3′-UTRs, exons, and 5′-UTRs, respectively. The most common GO term among these miRNA target genes with variants (integrity ≥ 0.7) was single-organism process and cellular process (Figure 3B).

We next analyzed the rare variants (MAF ≤ 0.05, integrity ≥ 0.7) in the miRNA target sites. A total of 106 rare SNPs and 30 rare INDELs were detected (Figure 2F). Approximately 33.50% of the variants were rare. Comparison of the rare variant ratio between miRNA target site and whole genome, we found miRNA target site had less rare variants. The 106 rare SNPs and 30 rare INDELs were linked to 124 miRNA target genes. The most commonly assigned GO term among these rare variant target genes was single-organism process (*P* < 0.05) (Figure 3C).

We subsequently focused on the SNPs affecting the complementation between miRNAs and their target genes. For example, the SNP A10C (A to C at the tenth nucleotide position) in the miR156–*SPL5* target site increased the target accessibility energy to unpair the target site (UPE) (Figure 2G); decreases in energy increase the likelihood that small RNAs will be able to interact with (and cleave) target mRNAs (Dai et al., 2018). Therefore, the SNP A10C might hinder the ability of miR156 to cleave *SPL5* in plants. The presence of two or more variants in one miRNA target site is another type of variation. Both G7A and T13C were within the miR5719-*NLR* target site (Figure 2G), which decreased the UPE from 14.456 to 12.499, thereby increasing the ability of miR5719 to bind and cleave *NLR* genes. Both *SPL5* and *NLR* genes are reportedly important for plant development and disease resistance (Ferreira e Silva et al., 2014; Shao et al., 2019; Jiang et al., 2020). Overall, specific variants that impair the binding of miRNAs, including A10C, G7A, and T13C, affect the expression and biological function of target genes.

### Phenotypes of 12 Important Agronomic Traits in Rapeseed

To reveal the possible biological effects of the variants in the miRNA target sites, we analyzed the correlation between the variants and rapeseed agronomic traits. We examined the following 12 important agronomic traits in 210 rapeseed accessions: plant height, oil content, branch angle, branch number, silique number, seed number, seed weight, ripening time, flowering time, cold resistance, stem lodging resistance, and *S. sclerotiorum* resistance (Figure 1C). The 210 rapeseed accessions were grown in the field in 2016, 2017, and 2018. The phenotypic variations among the accessions are summarized in Table 1. We then used one-way ANOVA to analyze the environmental effect variance to phenotypic variance. We found that seven traits over the 3 years showed no significant difference and the other five traits (branch angle, branch number, seed number, seed weight and *S. sclerotiorum* resistance) had at least 1 year showed significant difference (Supplementary Table 11). These results suggested that environmental factors in different years affected the above five traits. All phenotypic data collected in different environments had a normal or skewed distribution (Supplementary Figures 2A–C), suggesting the traits were quantitatively inherited.

**TABLE 1:** Phenotypic variations analysis among 210 *B. napus* accessions in the correlation panel.

Trait	Years	Min	Max	Median	Mean	SD	CV
Plant height (PH)	2016	53.00	207.60	156.05	151.91	25.23	0.17
	2017	54.25	257.60	145.00	142.05	22.84	0.16
	2018	69.83	252.20	136.57	136.30	23.36	0.17
Oil content (OC)	2016	26.29	51.41	43.35	42.81	4.53	0.11
	2017	29.43	49.00	40.17	40.16	3.97	0.10
	2018	27.72	50.07	39.86	39.68	4.12	0.10
Branch angle (BA)	2016	13.33	55.20	34.05	33.80	7.35	0.22
	2017	24.20	56.80	37.52	37.56	6.20	0.17
	2018	25.43	55.43	36.21	36.64	5.09	0.14
Branch number (BN)	2016	4.40	14.50	7.60	7.79	1.75	0.23
	2017	4.33	13.60	7.47	7.50	1.60	0.21
	2018	4.71	16.71	8.14	8.28	1.64	0.20
Silique number (SNPP)	2016	104.90	816.10	292.60	305.91	113.68	0.37
	2017	118.80	733.43	282.07	299.17	112.83	0.38
	2018	143.00	1324.50	318.86	339.47	137.10	0.40
Seed number (SNPS)	2016	10.42	36.70	23.20	23.15	4.49	0.19
	2017	7.80	27.45	19.08	18.57	4.41	0.24
	2018	11.02	37.68	24.40	24.39	5.33	0.22
Seed weight (SW)	2016	1.22	6.03	3.93	4.04	0.79	0.19
	2017	2.20	8.79	4.76	4.74	1.07	0.22
	2018	2.21	7.64	4.12	4.24	0.97	0.23
Ripening time (RT)	2016	223.00	248.00	234.00	233.95	3.99	0.02
	2017	235.00	259.00	243.00	243.21	4.56	0.02
	2018	234.00	260.00	246.00	246.98	4.67	0.02
Flowering time (FT)	2017	173.00	208.00	183.00	183.48	4.66	0.03
	2018	183.00	218.00	191.00	191.48	4.18	0.02

*CV: coefficient of variation; Max: Maximum; Min: Minimum; SD: Standard deviation.*

Next, we determined the pairwise correlations among the 12 traits (Supplementary Table 12). Silique number and branch number were the most positively correlated traits, whereas seed number and seed weight had the weakest negative correlation. The results of the correlation analysis imply that most of the traits were not independent of each other.

### Correlations Between Agronomic Traits and SNPs in miRNA Target Sites

To examine the correlations between the genetic variations in target sites and the phenotypic variance, we selected 199 high-quality SNPs (MAF > 0.05, integrity ≥ 0.7) in miRNA target sites for further analyses. We used the general linear model to clarify the correlations between the SNPs in miRNA target sites and the 12 important agronomic traits. A *P* value ≤ 0.005 (1/199 SNPs) was set as the threshold for the correlation study.

The single-SNP correlation approach revealed 223 (86 in 2016, 79 in 2017, and 58 in 2018) significant correlations between miRNA target sites and the examined phenotypes (*P* ≤ 0.005) (Supplementary Table 13). These significant SNPs partially explained the observed phenotypic variance (average *R*^2^ = 6.68, 6.69, and 7.34% in 2016, 2017, and 2018, respectively). The 223 correlated pairs involved 103 SNPs in 91 miRNA target genes. The relevant miRNA target genes included multiple transcription factor genes from the *MYB*, *NAC*, *SPL*, *NF-YA*, and *NBS-LRR* families. These transcription factors are related to various biological processes (e.g., growth and development and responses to abiotic or biotic stresses) in cells during the life cycle (He and Hannon, 2004; Ferreira e Silva et al., 2014; Si et al., 2016; Liu et al., 2020). These results indicate that the predicted candidate genes as well as the corresponding miRNAs might function together to regulate rapeseed growth and development.

Eleven significant SNP–trait correlations were consistently detected in the three consecutive years included in this study. According to the (contribution rate) *R*^2^ values, three SNPs (chrC04__46917368, chrA03__16353138, and chrC03__8380169) explained the phenotypic variance more than the other SNPs (Table 2). More specifically, chrC04__46917368, which was within the miR156–*SPL9* target site, explained 12.57–17.51% of the phenotypic variance in the three consecutive years. The SNP chrA03__16353138, which was correlated with the silique number and was located in the miR164–*CUC1* target site, explained 10.28–13.06% of the phenotypic variance. The SNP chrC03__8380169, which was correlated with the seed weight and was detected in the miR156–*SPL13* target site, explained 12.01–23.57% of the phenotypic variance. Considering they were detected in multiple years with high *R*^2^ values, the miRNA target genes *SPL9*, *CUC1*, and *SPL13* as well as the related miRNAs may contribute to branch, silique, and seed developmental processes, respectively.

**TABLE 2:** Co-detected SNPs significantly correlated with specific agronomic traits in three consecutive years.

Trait	SNP	−Log_10_(*P-value*)			Marker_*R*^2^	ALT	miRNA	Putative target genes	Homologous to *A. thaliana*	Annotation
		2016	2017	2018						
Branch angle	C04_46917368	8.82	6.89	9.58	12.57–17.51%	T/G	miR156/7	BnaC04g48150D	AT2G42200	Squamosa promoter binding protein-like 9
Branch number	C06_26535916	5.16	5.08	2.85	6.30–11.09%	T/A	miR395-3p	BnaC06g24960D	AT1G24190	SIN3-like 3
Oil content	A09_30690959	3.67	2.85	4.19	6.64–9.57%	C/T	miR169	BnaA09g44750D	AT1G18000	Major facilitator superfamily protein-2
Plant height	C03_7757082	2.60	4.99	5.33	6.51–12.89%	T/C	miR9559-5p	BnaC03g15500D	AT5G52320	Cytochrome P450%2C family 96%2C subfamily A%2C polypeptide 4
Plant height	A01_22873374	2.87	2.41	2.31	5.41–6.69%	T/G	miR1885-2	BnaA01g33710D	AT3G04210	Disease resistance protein (TIR-NBS class)-2
Plant height	A02_2884954	2.45	3.03	3.77	5.41–8.20%	G/A	miR165/6	BnaA02g06170D	AT5G60690	Homeobox-leucine zipper family protein/lipid-binding START domain-containing protein (REV)
Silique number	C03_8380169	2.79	3.08	2.79	4.80–5.39%	A/G	miR156/7	BnaC03g16490D	AT5G50670	Squamosa promoter-binding protein-like (SBP domain) transcription factor family protein
Silique number	A03_16353138	7.05	5.58	7.05	10.28–13.06%	A/C	miR164	BnaA03g33770D	AT3G15170	NAC (No Apical Meristem) domain transcriptional regulator superfamily protein
Seed weight	C03_8380169	7.07	12.68	6.07	12.01–23.57%	A/G	miR156/7	BnaC03g16490D	AT5G50670	Squamosa promoter-binding protein-like (SBP domain) transcription factor family protein
Seed weight	A07_random_468573	3.93	2.46	3.98	5.44–9.10%	G/A	miR393	BnaA07g36470D		
Seed weight	A03_14631943	3.88	2.90	2.85	6.39–8.40%	C/T	miR2111-5p	BnaA03g30140D	AT3G07680	Golgi-localized p24 protein

*R^2^ indicates the phenotypic variance explained by the SNP.*

Also, we investigated the linkage disequilibrium (LD) of each pair of SNPs, and performed GWAS for branch angle, silique number and seed weight using the mixed linear model (MLM), considering the genetic background, and found that the SNP of chrC04__46917368 (variation in *BnSPL9-3* targeted by miR156), chrA03__16353138 (variation in *BnCUC1-2* targeted by miR164) and chrC03__8380169 (variation in *BnSPL13-2* targeted by miR156) were in the loci associated with branch angle, silique number and seed weight, respectively (Supplementary Figure 3).

### Functional Analysis of the SNPs in miRNA Target Sites

**FIGURE 4:**
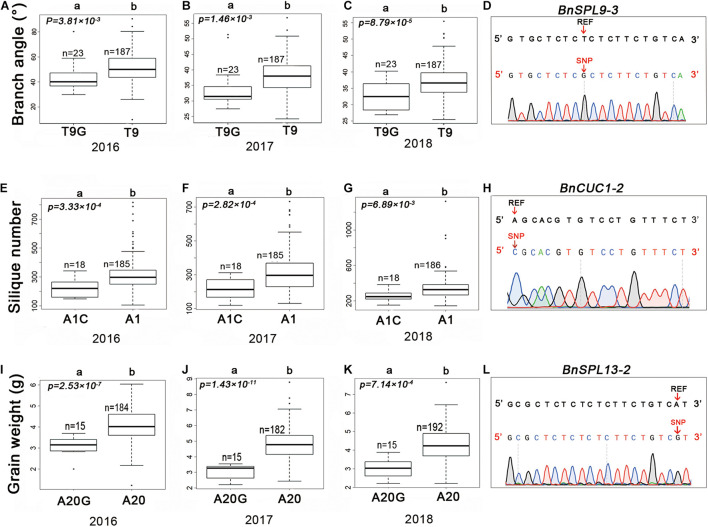
Haplotypes of candidate SNPs for the branch angle, silique number, and seed weight in rapeseed. Boxplots of the branch angle, silique number, and seed weight according to the SNP genotyping of *BnSPL9-3* **(A–C)**, *BnCUC1-2* **(E–G)**, and *BnSPL13-2* **(I–K)**. **(D,H,L)** Validation of variants in the candidate genes by Sanger sequencing. The variants are indicated by a red arrow. REF represents the reference sequence. Differences between the genotypes were analyzed using the *Wilcoxon rank-sum test* R package; “n” represents the number of accessions, whereas “a” and “b” represent significant differences at the 5% level.

A previous study proved that mutations in miRNA target sites in *A. thaliana* change the RNA abundance of the corresponding target genes (Mallory et al., 2004). To verify the effects of SNPs in the miRNA target sites on rapeseed agronomic traits, we first designed SNP-specific primers to validate the variants in miRNA target sites. The sequencing of the PCR products confirmed the authenticity of 98% of the target site variants. The three SNPs mentioned above were selected for further analyses. Consistent with the genotyping data, chrC04__46917368 was a T to G substitution (T9G) at the ninth target site nucleotide of *BnSPL9-3* (*BnaC04g48150D*) (Figure 4D). In contrast, chrA03__16353138 was an A to C substitution (A1C) at the first target site nucleotide of *BnCUC1-2* (*BnaA03g33770D*) (Figure 4H). The SNP chrC03__8380169 was an A to G substitution (A20G) at the twentieth target site nucleotide of *BnSPL13-2* (*BnaC03g16490D*) (Figure 4L).

We analyzed the effects of these three SNPs on agronomic traits in the 210 rapeseed accessions. The accessions with T9G in *BnSPL9-3* had smaller branch angles than the accessions with T9 (Figures 4A–C). The *BnCUC1-2* gene is a homolog of *AtCUC1*. An earlier study proved that knocking down *AtCUC* expression in *A. thaliana* affects silique development and results in abnormal pods (Liu et al., 2020). The accessions with A1C in *BnCUC1-2* produced fewer siliques than the accessions with A1 (Figures 4E–G). The *BnSPL13-2* gene is homologous to *OsSPL13*, which regulates seed size in rice (Si et al., 2016). The accessions with A20G in *BnSPL13-2* had a lower seed weight than the accessions with A20 (Figures 4I–K). According to the correlation analysis, these three SNPs were correlated with the observed phenotypic differences.

**FIGURE 5:**
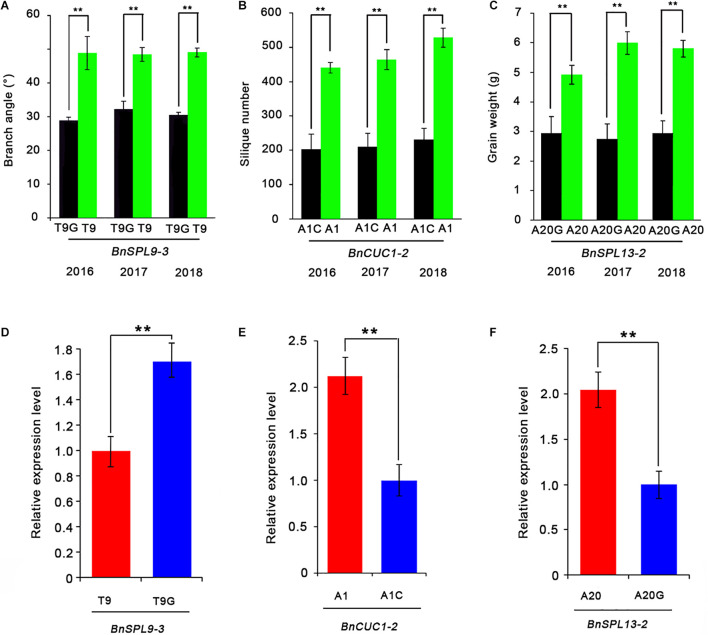
Effects of SNPs in the miRNA target site on target gene expression levels. **(A–C)** Effects of miRNA target site SNPs on the branch angle **(A)**, silique number **(B)**, and seed weight **(C)** determined by comparing groups with extremely high values (four accessions) and low values (four accessions) for these traits. **(D–F)** Comparison of the *BnSPL9-3* **(D)**, *BnCUC1-2* **(E)**, and *BnSPL13-2* **(F)** expression levels between the two groups with extremely different phenotypes. *ACTIN7* was used as an internal control for analyzing gene expression. Data are presented as the mean ± standard deviation (*n* = 3). Significant differences were determined by Student’s *t*-test: *P < 0.05, **P < 0.01.

Finally, to determine whether the SNPs in miRNA target sites affect gene silencing, we analyzed the expression levels of the miRNA-targeted genes in two groups of accessions with extremely different phenotypes by quantitative real-time PCR (qRT-PCR) (Figures 5A–C). Each group contained four accessions (Supplementary Table 3). The qRT-PCR data indicated the *BnSPL9-3* expression level was significantly higher in the accessions with T9G and the smallest branch angle than in the accessions with T9 and the largest branch angle (Figure 5D). The *BnCUC1-*2 expression level was significantly lower in the accessions with A1C and the fewest siliques than in the accessions with A1 and the most siliques (Figure 5E). The *BnSPL13-*2 expression level was significantly lower in the accessions with A20G and the lowest seed weight than in the accessions with A20 and the highest seed weight (Figure 5F). These results demonstrated that the three SNPs were functional and that a single SNP within the miRNA target sites was able to change target gene expression levels.

## Discussion

Mutations are a source of genetic variations. In many cases, genetic variations are related to phenotypic diversity. There are substantial genetic variations among crop populations. Additionally, previous studies revealed that sequence variations in mature and precursor miRNA sequences affect miRNA biogenesis (Ryan et al., 2010), but very little is known about the effects of genetic variations at target sites, especially in rapeseed. Our rapeseed population included 210 elite accessions collected from the major rapeseed-producing regions worldwide. All accessions were homozygous and suitable for genotyping and phenotyping. We re-sequenced the rapeseed population and detected millions of variants, of which 327 SNPs and 79 INDELs were in 357 miRNA-targeted sites. Hence, the genetic variation in the target sites is apparently extensive. Among the identified target site variants, most of the SNPs (80.12%) were in exons and 16.83% were located within UTRs (Supplementary Table 6), reflecting the fact the miRNAs targeted the 3′-UTR or the coding region of mRNAs.

Association analyses generally use common variants (MAF > 0.05) to detect significantly associated variants. The rare variants are eliminated from the genotypic data. However, in this study, we revealed that approximately 40.22% of the variants (SNPs and INDELs) in the whole genome were rare and 33.50% of the variants in the miRNA target sites were rare (MAF ≤ 0.05). The rare variants in a population have important but unknown roles influencing plant growth. The GO term enrichment analysis of the genes with these rare variants indicated that most were related to single-organism processes (Figure 3C). How the rare variants modulate phenotypes remains to be investigated.

Genetic variations at target sites can alter miRNA–mRNA interactions and affect the expression of miRNA target genes (Smith et al., 2015). The complementarity and thermodynamics of binding are critical factors related to the interaction between miRNAs and their target genes (Gong et al., 2012; Smith et al., 2015). Therefore, sequence variants, such as SNPs in the seed region or even in the cleavage site, may alter miRNA–mRNA interactions and affect the expression of miRNA target genes. Compared with animal miRNAs and their targets, the complementarity between plant miRNAs and their targets is greater and there are fewer mismatches in the binding region. It is possible that a SNP in the miRNA-targeted regions will disrupt gene regulation (Liu N. et al., 2014). The SNP A10C within the miR156–*SPL5* target site and the SNPs G7A and T13C within the miR5719–*NLR* target site were predicted to change the accessibility of the miRNA targets (Figure 2G). Accordingly, these SNPs might affect the expression and biological function of the target genes. Compared with prior studies that often focused on the change of protein coding genes, the large-scale detection of variants in miRNA target site could provide new clues for elucidating the miRNA-dependent gene expression regulation.

In this study, a SNP-based correlation analysis revealed 103 independent genetic variants, 223 significantly correlated pairs, and 91 miRNA target genes. Moreover, 11 SNP–trait correlations were detected in three consecutive years. Based on genome-wide association study using mixed linear model, we found the variation of miRNA target sites at *BnSPL9-3*, *BnCUC1-2*, and *BnSPL13-2* were in the loci associated with their correlated traits, respectively. However, due to the large LD block, these variations in miRNA target sites were not the only candidates in the trait loci, fine mapping were needed for further investigation. Notably, *BnSPL9-3*, *BnCUC1-2*, and *BnSPL13-2* expression levels varied between the accessions exhibiting extremely different phenotypes. A previous study demonstrated that the overexpression of zma-miR164e in transgenic *A. thaliana* downregulates *CUC1* expression (Liu et al., 2009). The consequences of this altered *CUC1* expression included the production of thin and short pods and the inability to produce normal pods and seeds, which ultimately seriously affected the number of pods per plant. The *BnSPL13-2* gene is a homolog of *OsSPL13*, which regulates seed size in rice (Si et al., 2016). These earlier studies further supported that *BnSPL13-2* may play the conserved role in controlling seed size. Therefore, these genes are worth considering to be used in crop improvement.

The miRNA target genes can also be miRNA biogenesis genes. For example, the AGO1 protein binds to miRNAs that post-transcriptionally repress target genes via the cleavage of mRNA and/or translational repression. The *AGO1* mRNA is targeted by miR168. Thus, variants in target sites might have important effects on miRNA biogenesis. A recent study in *B. rapa* indicated that a single-base variation (T to C) can alter the leaf hair phenotype (Zhang X. D. et al., 2018). Such SNPs are functional and can be used for designing molecular markers relevant for breeding (Zhang W. et al., 2018). In the current study, we identified a set of SNPs in miRNA target sites. Additionally, three of the SNPs (T9G, A1C, and A20G) in the target sites led to significant changes to the correlated traits in the rapeseed population (Figure 5). These SNPs may be candidate functional SNPs. In addition to the *B. napus* population, we also analyzed a *B. rapa* population (300 accessions) and a *B. oleracea* population (101 accessions) (NCBI accession number: PRJNA312457). Some SNPs in rapeseed miRNA target sites also exist in *B. oleracea* or *B. rapa* homologs, implying some of these genetic variations were derived from *B. oleracea* or *B. rapa*. Therefore, these SNPs appear to be conserved among *Brassica* crops and may contribute to the elite traits of *B. oleracea* or *B. rapa*.

Mutations in miRNA-targeted genes influence the domestication and selection of high-yielding and high-quality cultivars. A large branch angle is conducive to robust photosynthesis because of the increased exposure to sunlight. Increases in silique number and seed weight are crucial factors for high yields. Crop breeders have focused on the silique number and seed weight to improve their varieties. On the basis of the identification of SNPs in miRNA target sites, future investigations should explore the possibility of introducing specific mutations in miRNA target genes to enhance crop traits. This study generated a substantial amount of genotyping and phenotyping data for 210 rapeseed accessions, revealed the considerable genetic variations in miRNA target sites in rapeseed, and produced information regarding the correlations between SNPs in miRNA target sites and elite agronomic traits.

## Conclusion

In this study, through re-sequenced 210 elite rapeseed accessions, we identified large amount of variants in genome. Furthermore, we detected 330 SNPs and 79 INDELs in 357 miRNA target sites, some of which were rare variants. These variants possibly affect phenotypic variation. Three SNPs within the miRNA-binding regions of *BnSPL9-3*, *BnSPL13-2*, and *BnCUC1-2* were highly related to the branch angle, seed weight, and silique number, respectively. This outcome indicates that variation in the target sites is apparently extensive and distinctive. The genome-wide genetic variations in miRNA-targeted sites provide researchers and breeders a choice to detecting the allelic variation in miRNA target site related to elite traits.

## Data Availability Statement

The datasets presented in this study can be found in online repositories. The names of the repository/repositories and accession number(s) can be found below: NCBI (accession: PRJNA748869).

## Author Contributions

PX conceived the project. PX and YH designed the experiments. PX and YtZ performed the experiments. YfZ, JJ, LY, and JM analyzed and interpreted the data. PX, XY, and YH wrote the manuscript. All authors contributed to the article and approved the submitted version.

## Conflict of Interest

The authors declare that the research was conducted in the absence of any commercial or financial relationships that could be construed as a potential conflict of interest.

## Publisher’s Note

All claims expressed in this article are solely those of the authors and do not necessarily represent those of their affiliated organizations, or those of the publisher, the editors and the reviewers. Any product that may be evaluated in this article, or claim that may be made by its manufacturer, is not guaranteed or endorsed by the publisher.

## Footnotes

1
http://brassicadb.cn/

2
https://github.com/broadinstitute/gatk

3
https://hpc.nih.gov/apps/ANNOVAR.html

4
https://github.com/NBISweden/AGAT

5
https://www.zhaolab.org/psRNATarget/

6
https://www.maizegenetics.net/tassel

7
https://github.com/BGI-shenzhen/PopLDdecay

8
http://www.mirbase.org/

9
https://www.arabidopsis.org

## Supplementary Material

Supplementary Figure 1 Comparison of protein-coding gene and miRNA target site sequences in *B. napus* and *A. thaliana*. **(A)** Comparison of protein-coding genes between *B. napus* and *A. thaliana*. **(B)** Putative target sites. **(C)** Target genes with sequence variations in *B. napus*.

Supplementary Figure 2 Frequency distribution of the 12 examined agronomic traits in rapeseed in three consecutive years. **(A–C)** Frequency distribution of 12 traits in 2016 **(A)**, 2017 **(B)**, and 2018 **(C)**. PH: plant height; OC: oil content; BA: branch angle; BN: branch number; SNPP: silique number; SNPS: seed number; SW: seed weight; RT: ripening time; FT: flowering time; RC: cold resistance; RSL: stem lodging resistance; RS: *Sclerotinia sclerotiorum* resistance.

Supplementary Figure 3 Genome-wide association studies of branch angle, silique number and seed weight. Local Manhattan plot (top) and LD heatmap (bottom) surrounding the SNP. Arrow indicates the position of chrC04__46917368 **(A)**, chrA03__16353138 **(B)**, and chrC03__8380169 **(C)**. The local Manhattan was shown of the SNPs in flanking region (−1 Mb to +1 Mb) of the targeted sites. LD heatmap of SNPs in the qSPL9 **(D)**, qCUC1 **(E)**, and qSPL13 **(F)** loci, respectively. The local LD was calculated of the SNPs in flanking region (−1 kb to +1kb) of the targeted sites.

Supplementary Table 1 Summary of all accessions sequenced in this study.

Supplementary Table 2 Primers used in this study.

Supplementary Table 3 Accessions analyzed by qRT-PCR.

Supplementary Table 4 Software and websites used in this study.

Supplementary Table 5 Number of non-redundant reads for each accession.

Supplementary Table 6 Annotation of the SNPs and INDELs identified in *B. napus*.

Supplementary Table 7 Crucifer family miRNA sequences.

Supplementary Table 8 Putative miRNA target site sequences in rapeseed transcripts.

Supplementary Table 9 Global annotation of protein-coding genes in *B. napus*. The BLAST algorithm was used to compare the *Arabidopsis thaliana* transcripts with *B. napus* transcripts (threshold: e-50).

Supplementary Table 10 SNPs and INDELs in miRNA target sites in rapeseed accessions.

Supplementary Table 11 Analysis of variance of the 12 observed agronomic traits. Significant differences were determined by *F*-test (ANOVA) using the SAS software. The letter “A” and “B” represent significant differences at the 1% level.

Supplementary Table 12 Correlations among the 12 observed agronomic traits. The correlation analysis of the different traits was performed using the *cor.test* function in R (version 3.6.1). Significance levels: ^∗^*P* < 0.05; ^∗∗^*P* < 0.01; ^∗∗∗^*P* < 0.001.

Supplementary Table 13 Summary of significantly correlated SNPs.
