# Supplementary material for: Global Analysis of the Genetic Variations in miRNA-Targeted Sites and Their Correlations With Agronomic Traits in Rapeseed
Source: Front Genet. 2021 Sep 14;12:741858. doi: 10.3389/fgene.2021.741858 (PMC8476912; doi:10.3389/fgene.2021.741858)
Supplement: Supplementary Figure 1 — Comparison of protein-coding gene and miRNA target site sequences in B. napus and A. thaliana. (A) Comparison of protein-coding genes between B. napus and A. thaliana. (B) Putative target sites. (C) Target genes with sequence variations in B. napus. [file Data_Sheet_2.docx]

Supplementary Material

**Global analysis of the genetic variations in miRNA-targeted sites and their correlations with agronomic traits in rapeseed**

**Pengfei Xu^1, 2,^** ^†^**, Yantao Zhu^3,^** ^†^**, Yanfeng Zhang^3,^** ^†^**, Jianxia Jiang^4^, Liyong Yang^4,^*, Jianxin Mu^3,^*, Xiang Yu^5,^* and Yuke He^1, 2,^***

^1^National Key Laboratory of Plant Molecular Genetics, CAS Center for Excellence in Molecular Plant Sciences, Shanghai Institute of Plant Physiology and Ecology, Chinese Academy of Sciences, Shanghai 200032, China

^2^University of the Chinese Academy of Sciences, Beijing 100049, China

^3^Hybrid Rape Research Center of Shaanxi Province, Yangling, Shaanxi 712100, China

^4^Crop Breeding and Cultivation Research Institute, Shanghai Academy of Agricultural Sciences, Shanghai 201403, China

^5^School of Life Sciences and Biotechnology, Shanghai Jiao Tong University, Shanghai 200240, China

^†^These authors have contributed equally to this work and share first authorship.

*** Correspondence:**Yuke He
[heyk@sippe.ac.cn](mailto:heyk@sippe.ac.cn)

Xiang Yu
[yuxiang2021@sjtu.edu.cn](mailto:yuxiang2021@sjtu.edu.cn)

Jianxin Mu
[jxmsxyc@163.com](mailto:jxmsxyc@163.com)

Liyong Yang
[yangliyong@saas.sh.cn](mailto:yangliyong@saas.sh.cn)

## Supplementary Figures


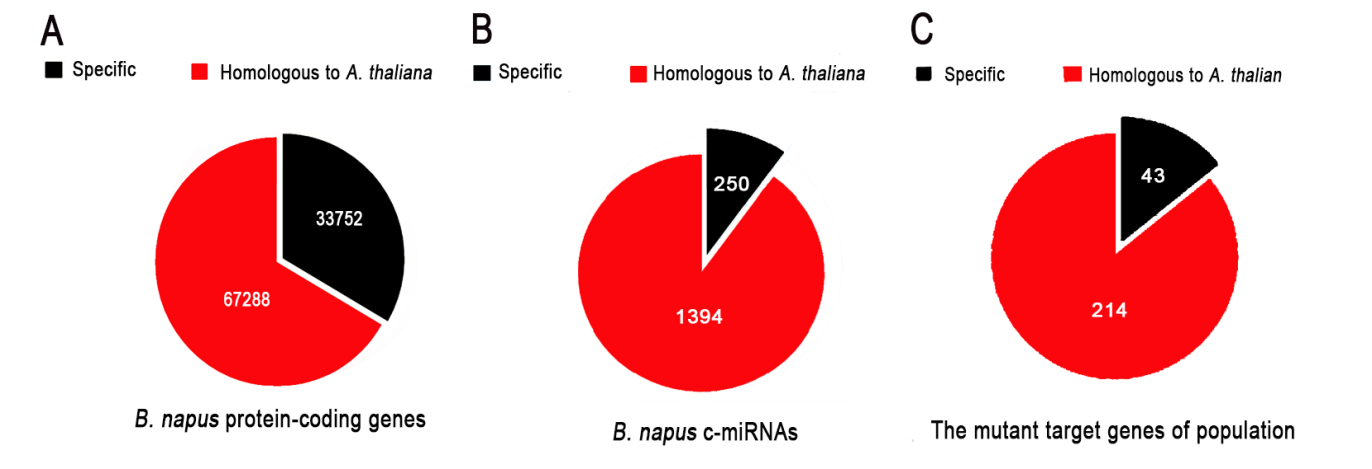


**Supplementary Figure 1.** Comparison of protein-coding gene and miRNA target site sequences in *B. napus* and *A. thaliana*. **(A)** Comparison of protein-coding genes between *B. napus* and *A. thaliana*. **(B)** Putative target sites. **(C)** Target genes with sequence variations in *B. napus*.


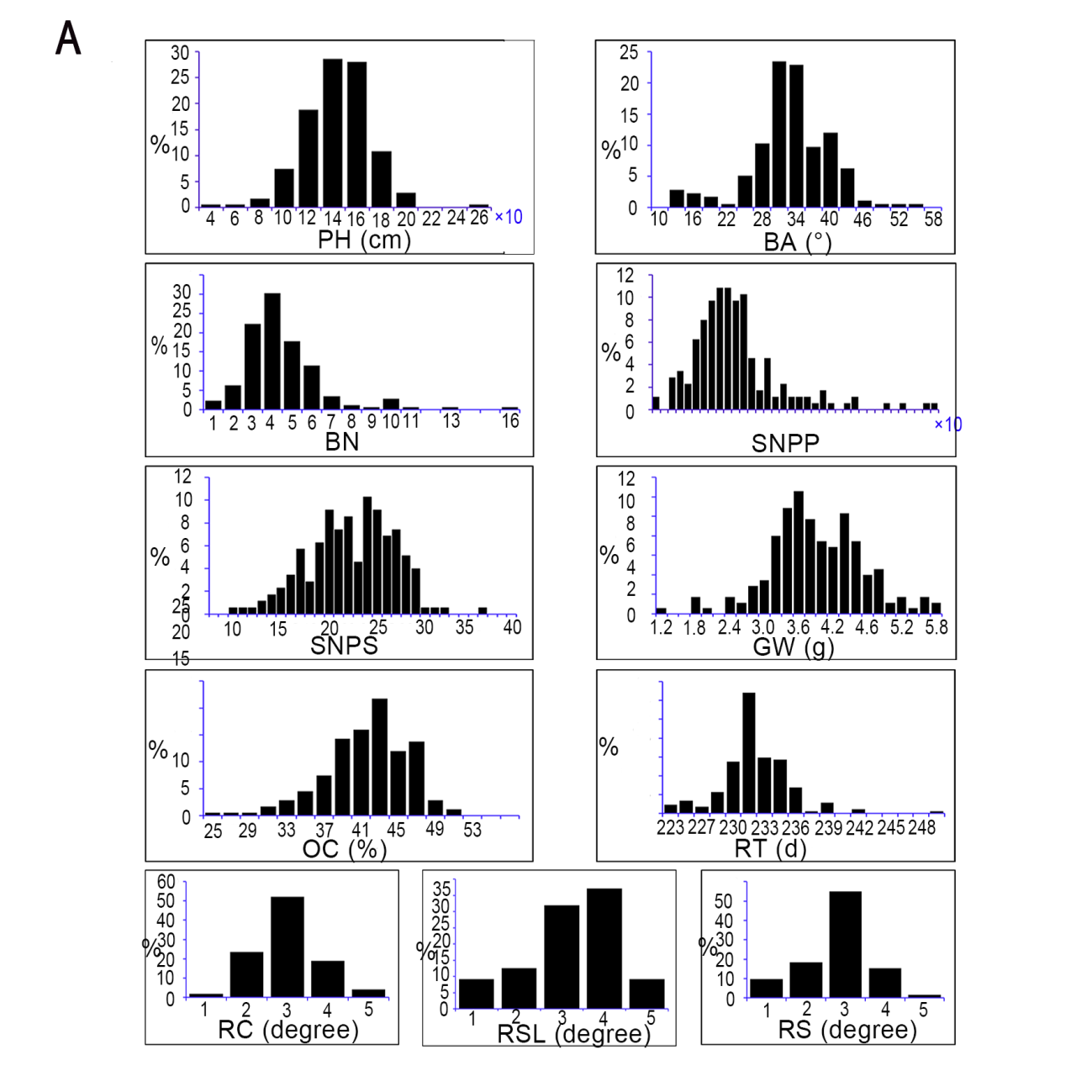


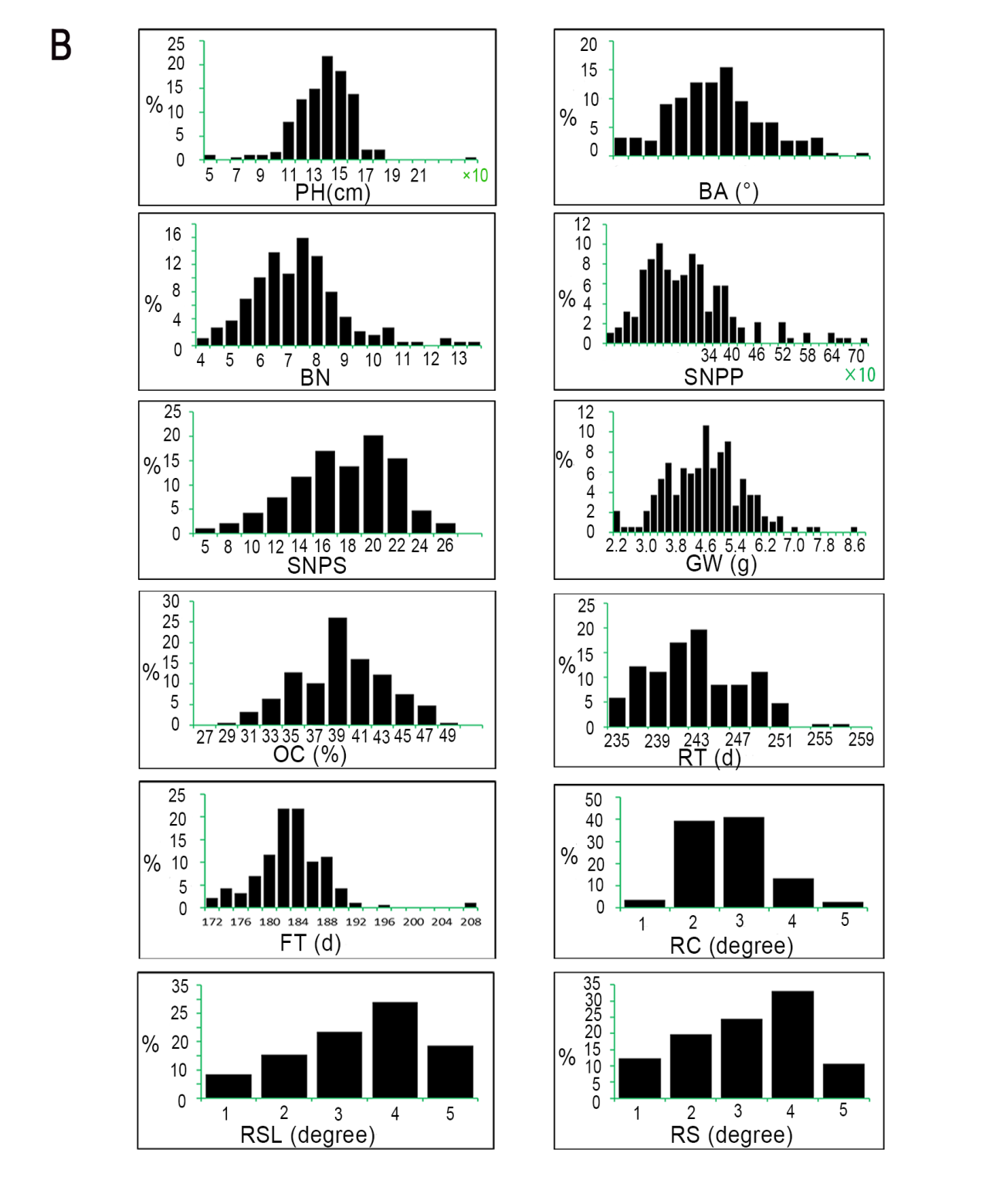


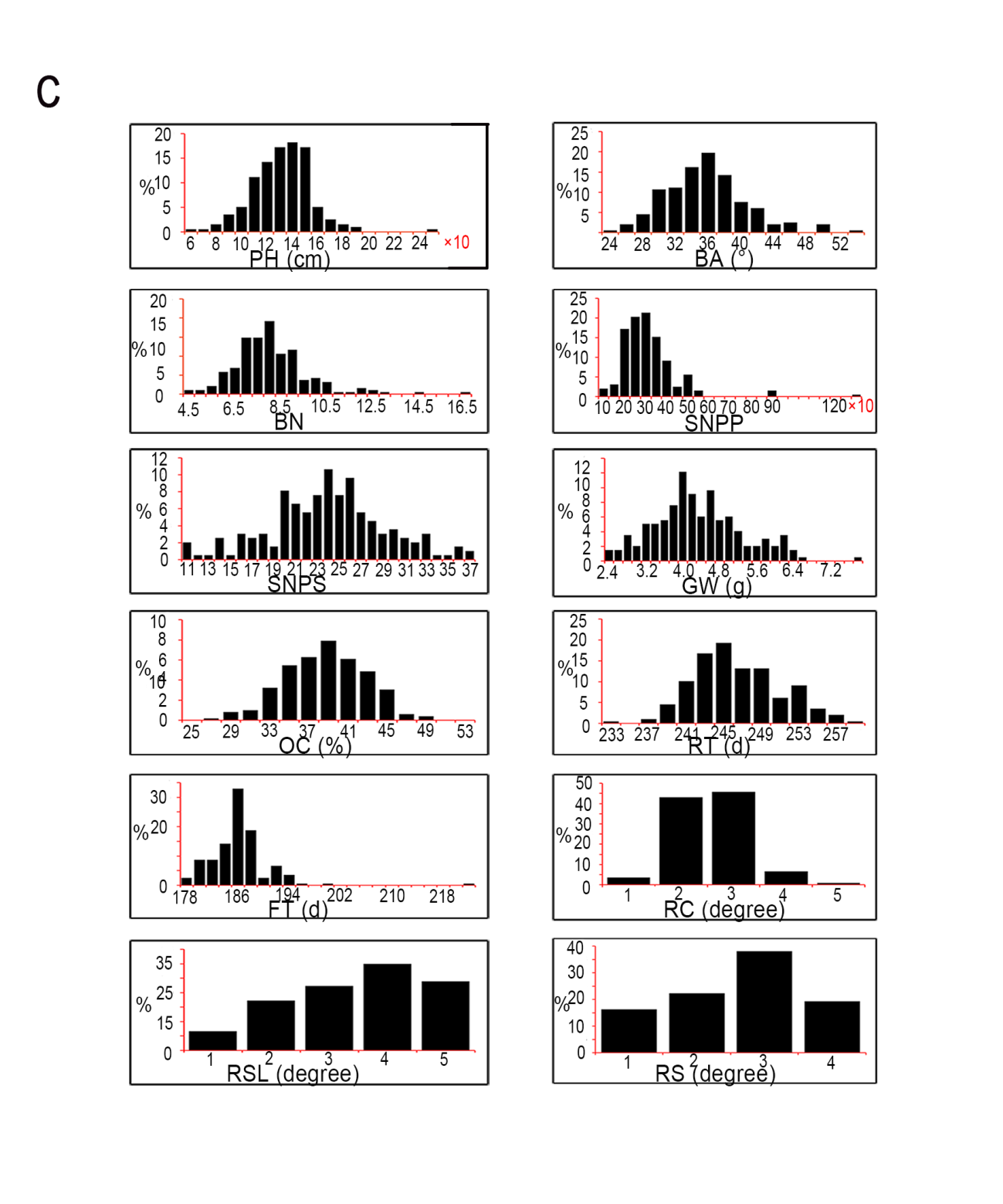


**Supplementary Figure 2.** Frequency distribution of the 12 examined agronomic traits in rapeseed in 3 consecutive years. **(A–C)** Frequency distribution of 12 traits in 2016 **(A)**, 2017 **(B)**, and 2018 **(C)**. PH: plant height; OC: oil content; BA: branch angle; BN: branch number; SNPP: silique number: SNPS: seed number; SW: seed weight; RT: ripening time; FT: flowering time; RC: cold resistance; RSL: stem lodging resistance; RS: *Sclerotinia sclerotiorum* resistance.


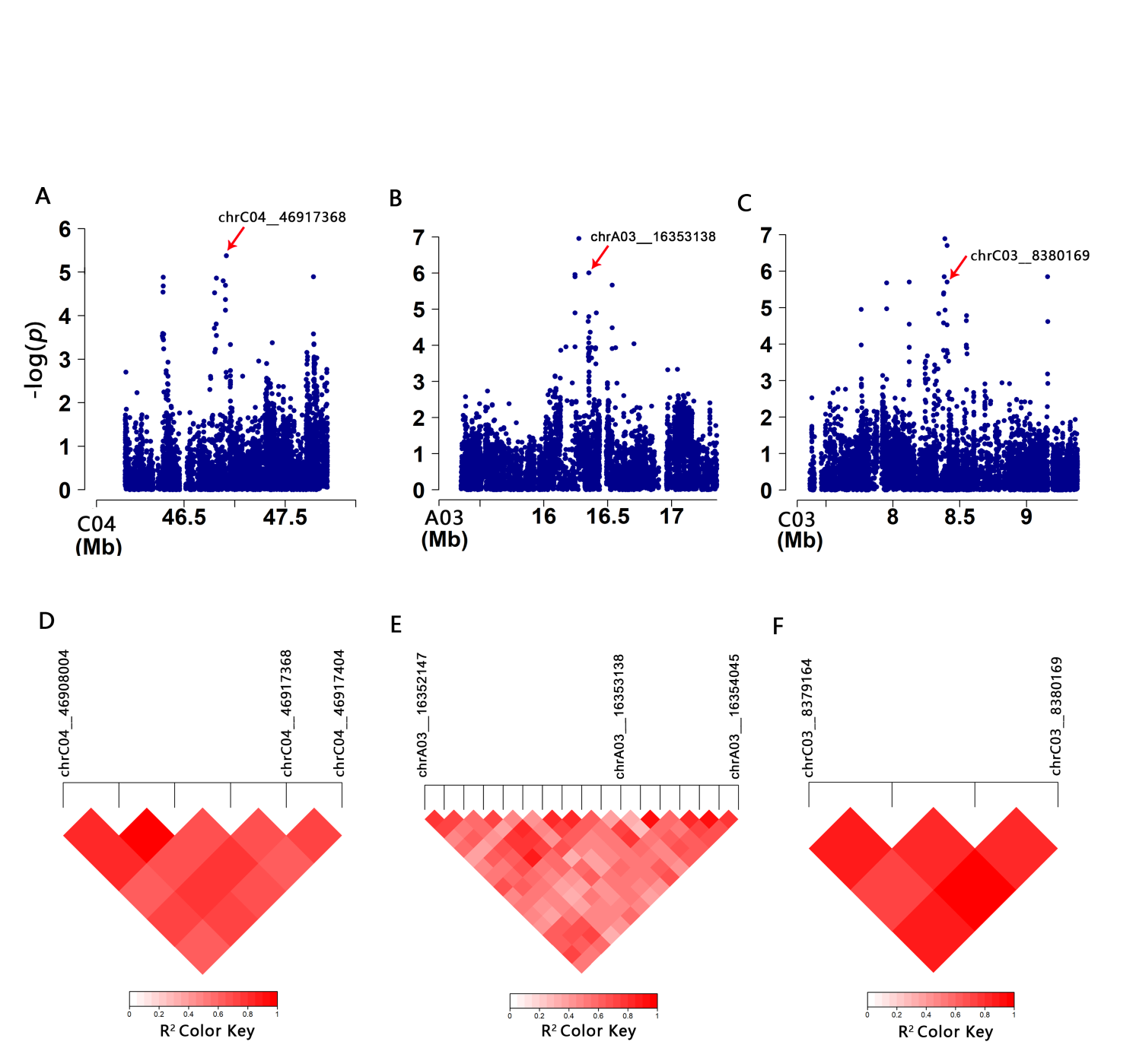


**Supplementary Figure 3**. Genome-wide association studies of branch angle, silique number and seed weight. Local Manhattan plot (top) and LD heatmap (bottom) surrounding the SNP. Arrow indicates the position of chrC04__46917368 (**A**), chrA03__16353138 (**B**) and chrC03__8380169 (**C**). The local Manhattan was shown of the SNPs in flanking region (-1Mb to +1Mb) of the targeted sites. LD heatmap of SNPs in the qSPL9 **(D),** qCUC1 **(E)** and qSPL13 **(F)** loci. The local LD was calculated of the SNPs in flanking region (-1kb to +1kb) of the targeted sites.
